# Sarcoid Here, Sarcoid There, Sarcoid Everywhere

**DOI:** 10.7759/cureus.34904

**Published:** 2023-02-12

**Authors:** Peter A Iskander, Preya Patel, Ronakkumar Patel, Chilsia Shafi, Jiayi Zheng, Anthony Iskander, Jacob Miller

**Affiliations:** 1 Internal Medicine, The Wright Center for Graduate Medical Education, Scranton, USA; 2 Internal Medicine, Xavier University School of Medicine, Oranjestad, ABW; 3 Internal Medicine, Wilkes-Barre VA Medical Center, Scranton, USA

**Keywords:** hypocalcemia, anemia, renal cell carcinoma, extrapulmonary manifestations, sarcoidosis

## Abstract

Although usually more associated with the lungs, sarcoidosis can have multiple extrapulmonary manifestations. We present a case of a patient with previous biopsy-proven sarcoidosis who was admitted to the hospital secondary to worsening shortness of breath. The patient was found to be positive for Respiratory Syncytial Virus (RSV) which was believed to have exacerbated his pulmonary symptoms. He was treated with IV steroids, nebulizers, and antibiotics which ultimately helped relieve his symptoms. In terms of his sarcoidosis, he was previously treated in the past with steroids in regards to this pathology (which is the mainstay of treatment); while on the regimen, the patient noted his breathing was improved. Of note, he did also have a history of renal cell carcinoma (RCC) status post nephrectomy which was initially evaluated for possible sarcoidosis involvement. This medical therapy could also have been the reason his sarcoidosis did not progress to involve other organs.

## Introduction

Although typically associated with respiratory complications, sarcoidosis can affect many other organs; symptoms can mimic each other and so can lead to deviations in diagnosis paths. With its characteristic noncaseating granulomas, it is more typically seen in African American females [1]. In one US study from 2010-2013, it was noted that incidence and prevalence were significantly higher in African Americans when compared to the White, Hispanic, or Asian population [2]. Knowledge of proper suspicious findings is important in patient workup to help differentiate sarcoidosis manifestations (whether it be pulmonary or extrapulmonary) with other pathologies as the treatment regimen path can vary vastly. Good history taking, physical findings, laboratory tests, and proper imaging are all crucial parts in helping lead to the most accurate diagnosis.

## Case presentation

A 56-year-old male with a past medical history of renal carcinoma status post nephrectomy, hypertension, and IV drug use presented to the emergency room with complaints of progressively increasing shortness of breath over a period of four days. Symptoms were associated with acute onset of yellowish sputum production. The patient denied any fevers, weight loss, or chest pain. He was an active smoker with a 40-pack-year history. Lab work was remarkable for elevated troponins and positive Respiratory Syncytial Virus (RSV) testing; the remainder was within normal limits. CT chest noted multiple pulmonary nodules in the mediastinal, subcarinal and paratracheal lymph nodes, and a 4.5 cm upper pole right kidney density with increased area of calcification. He was admitted for an acute chronic obstructive pulmonary disease (COPD) exacerbation thought to be triggered by the RSV virus. He was initiated on IV methylprednisolone, Duonebs, and Doxycycline. Prior to this, in 2007 he had a granuloma on the left arm which was thought to be secondary to a foreign body as the pathology had talc and cotton, and he was an IV drug user. Of note, in 2008 he underwent Endobronchial Ultrasound (EBUS) with pathology-proven granulomatous disease; he was prescribed a short duration of prednisone in May of 2008 following which he had modest improvement in his Forced Expiratory Volume (FEV1) as well as his symptoms. Upon cessation of steroid therapy, however, he quickly returned to his diminished baseline respiratory function. Additionally, during his hospital stay, the patient had multiple episodes of symptomatic bradycardia for which Cardiology was consulted; Zio-patch was placed on discharge and he was recommended to undergo further screening for cardiac involvement of his sarcoidosis.

## Discussion

Laboratory testing

**Figure 1:**
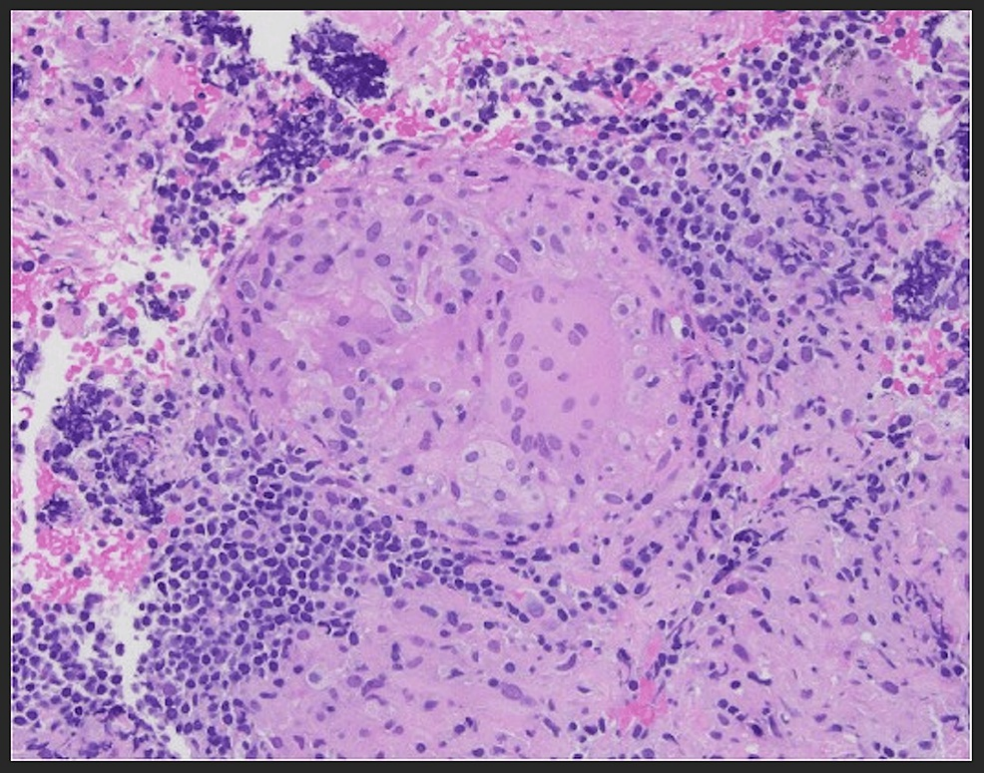
Image depicting a lymph node biopsy of a noncaseating granuloma

Sarcoidosis is a disease that can affect multiple organs and so various workup and diagnostic testing must be performed to help differentiate it from other similar presenting pathologies. Diagnosis can be narrowed down via imaging, symptoms, biopsy-proven noncaseating granulomas, as well as exclusion of other pathology/malignancy (Figure 1) [3]. Someone presenting with unexplained shortness of breath, constitutional symptoms and hilar lymphadenopathy, for example, should raise suspicion for sarcoid pathology and warrant further investigation [4].

Bronchoalveolar lavage (BAL) can be a useful tool in differentiating sarcoidosis from other lung pathologies. In one study that compared BAL fluid analysis of healthy lungs to those of symptomatic untreated sarcoidosis patients, it was noted that the lymphocyte count, CD4/CD8 ratio, as well as total cell count were all elevated [5].

Angiotensin-converting enzyme (ACE) is also a marker of disease severity. Although also elevated in other granulomatous diseases, ACE levels in patients with sarcoidosis are correlated to the severity of whole-body granuloma presence [6]. These levels can therefore be used to monitor for disease improvement vs progression during treatment.

Imaging

With regards to imaging, chest radiography is the most common modality utilized and can help with the initial staging (0-4) based off criteria such as bilateral hilar lymphadenopathy and the presence of fibrosis (Table 1) [7].

**Table 1:** Table indicating progressive radiologic staging of sarcoidosis

Stage Number	Radiographic Findings
0	Normal Radiograph
1	Bilateral hilar lymphadenopathy
2	Bilateral hilar adenopathy with parenchymal infiltrates
3	Parenchymal infiltrates only
4	Pulmonary fibrosis

CT and MR imaging can also be utilized to help further investigate the extent of disease, especially when evaluating for extrapulmonary spread; they are more sensitive in detecting parenchymal pathology and lymphadenopathy. CT in particular can be useful for guidance when trying to obtain biopsy specimens as well [8].

Management

At this time treatment of asymptomatic sarcoidosis is not currently indicated; those with pulmonary and extrapulmonary manifestations, however, do warrant further management.

Although there is no official guideline regarding treatment, current therapy recommendations involve IV vs oral corticosteroids based on disease severity. They have been shown to be an effective management strategy, especially in patients with sarcoid-induced renal impairment [9]. In a study by Naderi et al. patients diagnosed with sarcoid underwent kidney biopsy; all patients were noted to have kidney failure secondary to interstitial nephritis with and without granulomas. After a mean follow-up of 59 months, they noted that Prednisolone 0.5 mg/kg seemed to be a sufficient dose to achieve remission [10]. In those whose disease is refractory to steroid treatment, various biologics can be utilized in conjunction.

Subgroups

Löfgren syndrome is one disease associated with sarcoidosis. Its pathology can be indicated by the acuteness of symptoms when compared to the more chronic ones seen in general sarcoidosis. Other manifestations can include Erythema Nodosum, arthritis, constitutional symptoms, as well as similar hilar lymphadenopathy. The overall prognosis is generally noted to be better in this subgroup [11].

Heerfordt-Waldenström syndrome is another rare presentation. It can be characterized by its neurologic involvement. Patients with this subgroup can present with facial nerve palsy, parotid gland involvement, as well as uveitis [12]. These manifestations are likely due to the granulomatous presence secondary to the underlying sarcoidosis. Granulomas within the neural fibers in the face can lead to inflammation and impingement causing weakness. Those that develop within the parotid glands can lead to nearby infections or even difficulty swallowing secondary to mass effect and local swelling/inflammation. Finally, with regards to the eyes, uveitis can develop causing pain, erythema, and blurriness of vision.

Extrapulmonary manifestations

With regards to renal manifestations, sarcoidosis can present in a number of different ways including electrolyte imbalances, masses, nodules, etc. These can therefore be confused with primary renal pathologies or malignancies, for which our patient did have history of [13].

Due to the granuloma formation associated with the disease, there is an activation of enzymes; predominantly alpha-1-hydroxylase. The overall effect is the net conversion of inactive 25-hydroxyvitamin D to its active form of 1,25 Hydroxyvitamin D; this can lead to an increase in calcium absorption causing hypercalcemia and hypercalciuria [14]. Elevated levels of calcium can lead to nephrolithiasis and nephrocalcinosis which can cause kidney damage [10]. If remained untreated, this can progress to end-stage renal disease requiring hemodialysis [15].

Rhythm issues in patients with known sarcoidosis can warrant evaluation for possible cardiac involvement. As with our patient, he did have multiple episodes of symptomatic bradycardia when hospitalized. Other known manifestations of cardiac sarcoidosis involve heart block, chest pain, arrhythmias, and shortness of breath; the latter of which the patient also endorsed and was hospitalized for [16]. Further workup to include EKG, echocardiograms, and evaluation for ICD should be pursued.

Anemia is also another manifestation that can be due to the formation of non-caseating granulomas produced in the bone marrow and kidneys. Renal cell carcinoma (RCC) can also present with similar findings. This can be due to the involved kidney damage from the RCC causing decrease in erythropoietin release [17]. The incidence of RCC has steadily been increasing every year, roughly 2-4% per year, and has one of the highest mortality rates of genitourinary malignancies [18]. Only roughly 10% present with the “classic triad” of hematuria, flank pain, and palpable mass [19]. These manifestations can mimic those with sarcoidosis who have renal involvement. Sarcoid-like granulomatous reactions can also be seen in various malignancies and so careful workup needs to be completed to be able to differentiate the etiology of the granulomas [20].

## Conclusions

Sarcoidosis can have vague presentations when affecting the renal or other system and so its involvement can potentially be overlooked. Similar manifestations (such as anemia, flank pain/mass, electrolyte imbalances) can mimic other pathologies. Granulomas, in particular, can be of concern as their etiology can be from the sarcoidosis or various other reasons. It is then crucial that proper workup be done to be able to differentiate it, as the management can be very different; possible nephrectomy in those with RCC, for example, as opposed to steroid management with sarcoid-induced kidney injury. Mortality from sarcoidosis has been slowly rising, but early interventions and younger diagnosis age can be good prognostic factors that can help significantly decrease the risk of death and long-term complications.
